# Supplementary figures and images for: Unveiling dynamic metabolic signatures in human induced pluripotent and neural stem cells
Source: PLoS Comput Biol. 2020 Apr 16;16(4):e1007780. doi: 10.1371/journal.pcbi.1007780 (PMC7188302; doi:10.1371/journal.pcbi.1007780)

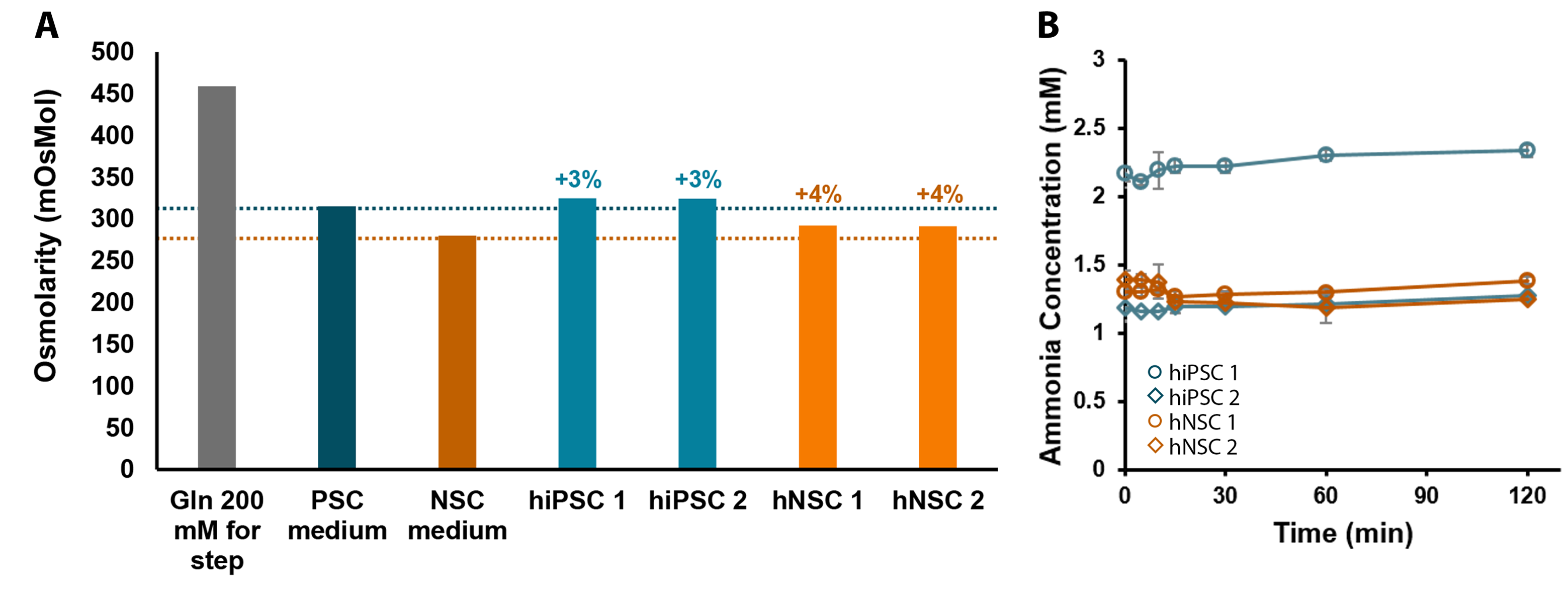

Supplement: S1 Fig — (A) Osmolarity of the solution of glutamine used for the perturbation step, of the cell culture media used for hiPSC and hNSC and of the culture media of the four bioreactor cultures immediately after the glutamine perturbation step. Changes in osmolarity after the perturbation step are indicated in percentage on top of each bar. (B) Ammonia concentration in bioreactors culture media. (TIF) [file pcbi.1007780.s001.tif]

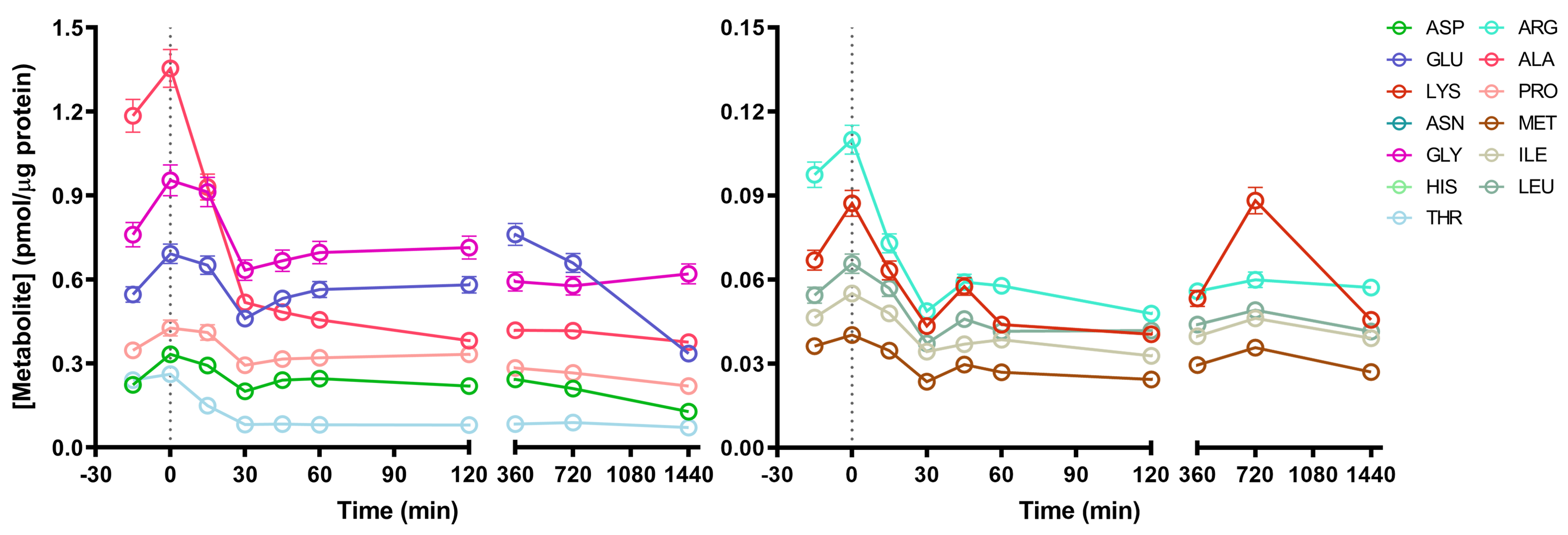

Supplement: S2 Fig — Metabolic profiles of alanine and threonine in an experiment covering up to 24 hours after the glutamine step increase demonstrate that 2 hours is usually sufficient for reaching a new metabolic steady-state. (TIF) [file pcbi.1007780.s002.tif]

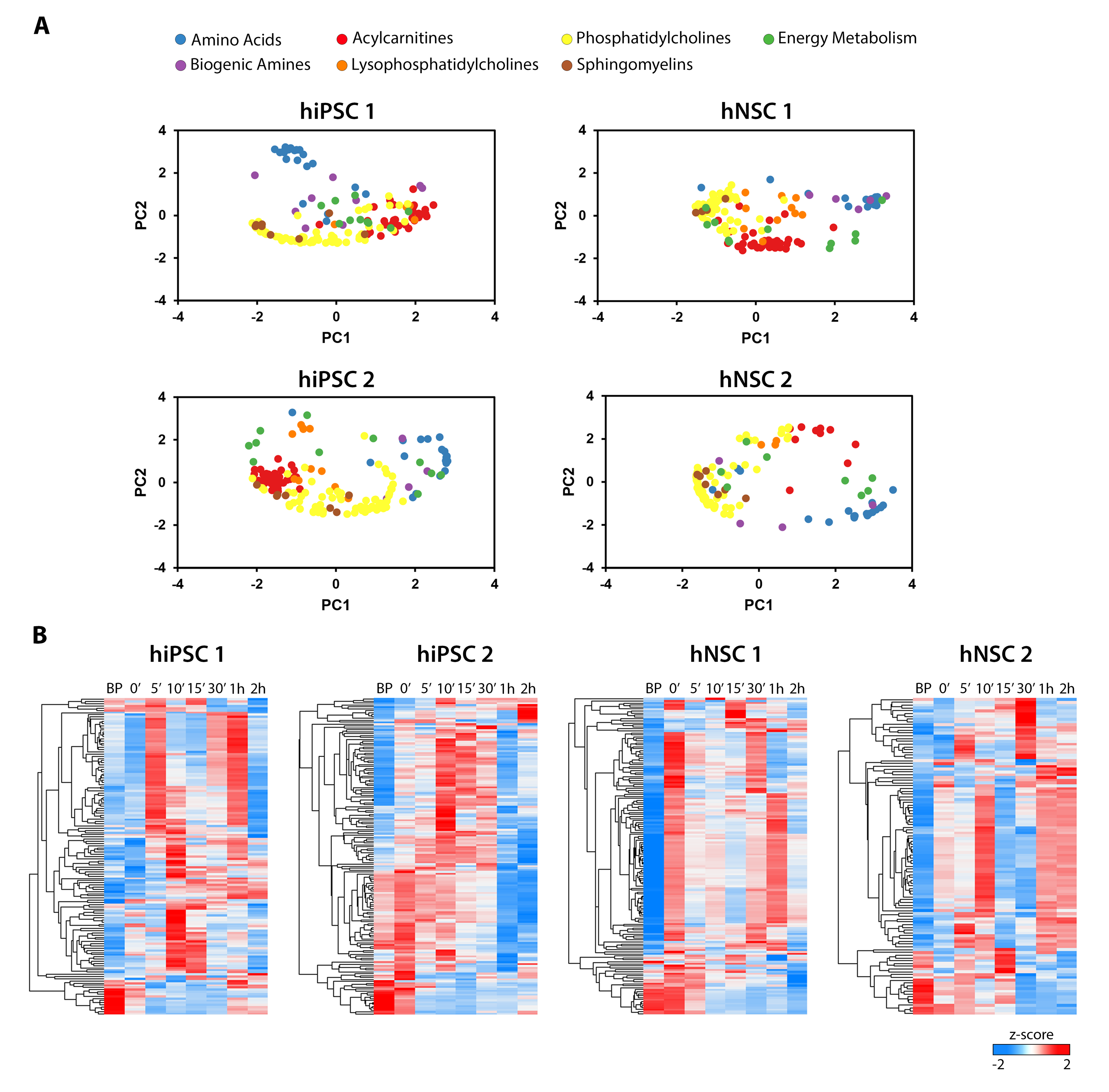

Supplement: S3 Fig — The dynamic profiles of molar quantities per protein were normalized by a z-score procedure (see Materials and Methods). (A) Principal component analysis of metabolic profiles. (B) Hierarchical clustering of metabolic profiles. Rows represent the different metabolites, while each column represents one time point (BP–before pulse, 0, 5, 10, 15, 30 min, 1, 2 hours). (TIF) [file pcbi.1007780.s003.tif]

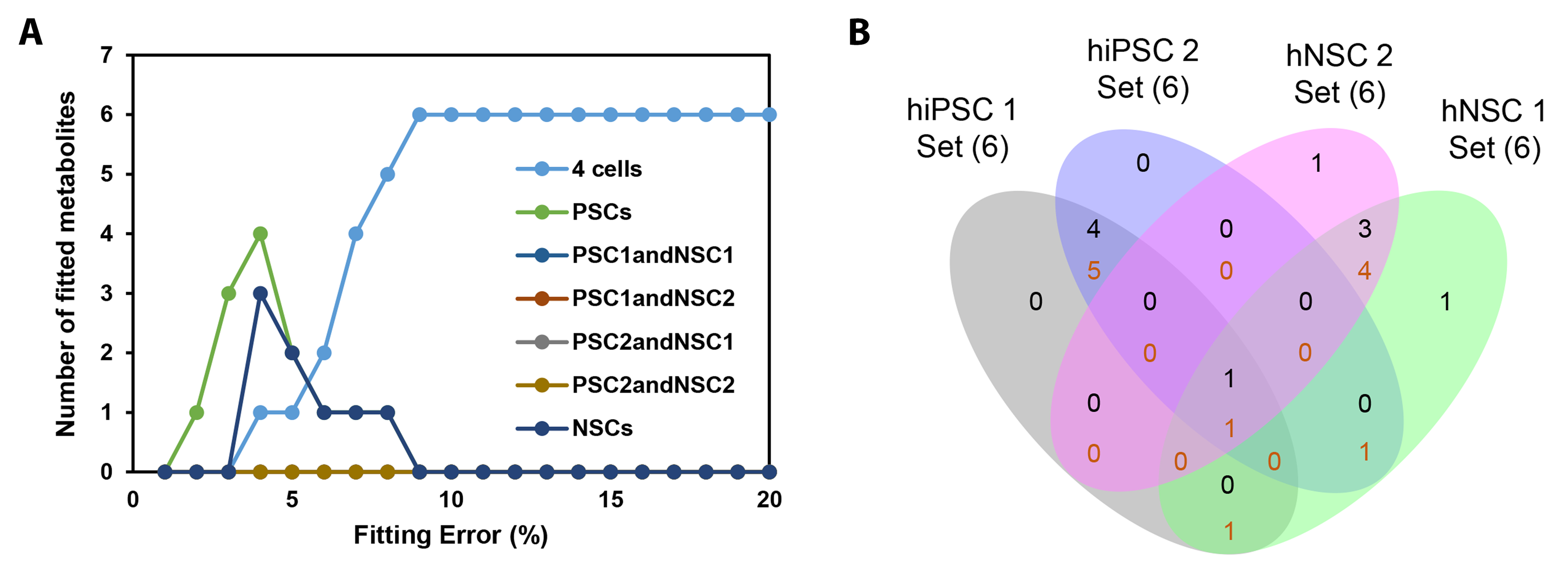

Supplement: S4 Fig — (A) Frequency of fitted metabolites along the threshold of the fitting error, to several combinatorial groups of cells. (B) Venn diagram of metabolites, present in all four cell lines, with fits below a 4% error to all cell types. Orange numbers indicate the number of all simulated metabolic profiles that fit to that region, regardless of fitting to other regions with the same or higher number of intersections. (TIF) [file pcbi.1007780.s004.tif]

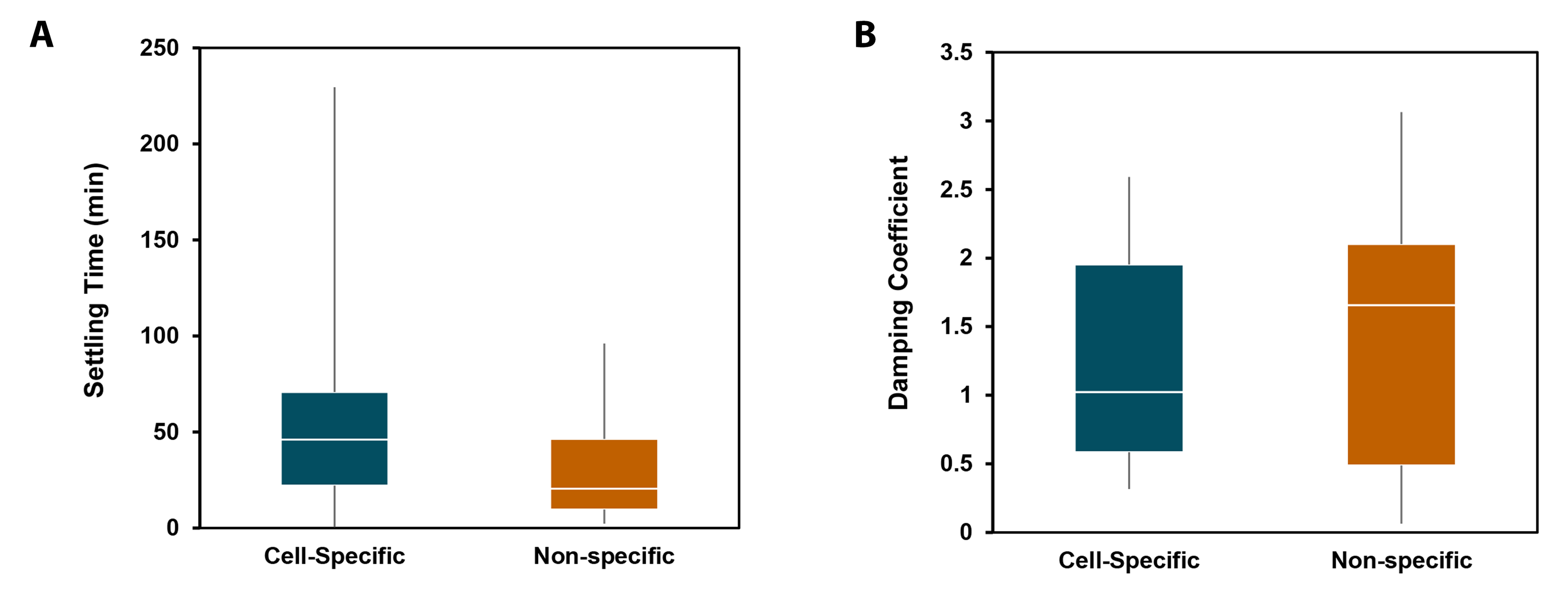

Supplement: S5 Fig — (A) Boxplot of settling time of simulated metabolic profiles between cell type-specific and shared dynamics (non-specific). (B) Boxplot of damping coefficient of simulated metabolic profiles between cell type-specific and shared dynamics (non-specific). (TIF) [file pcbi.1007780.s005.tif]

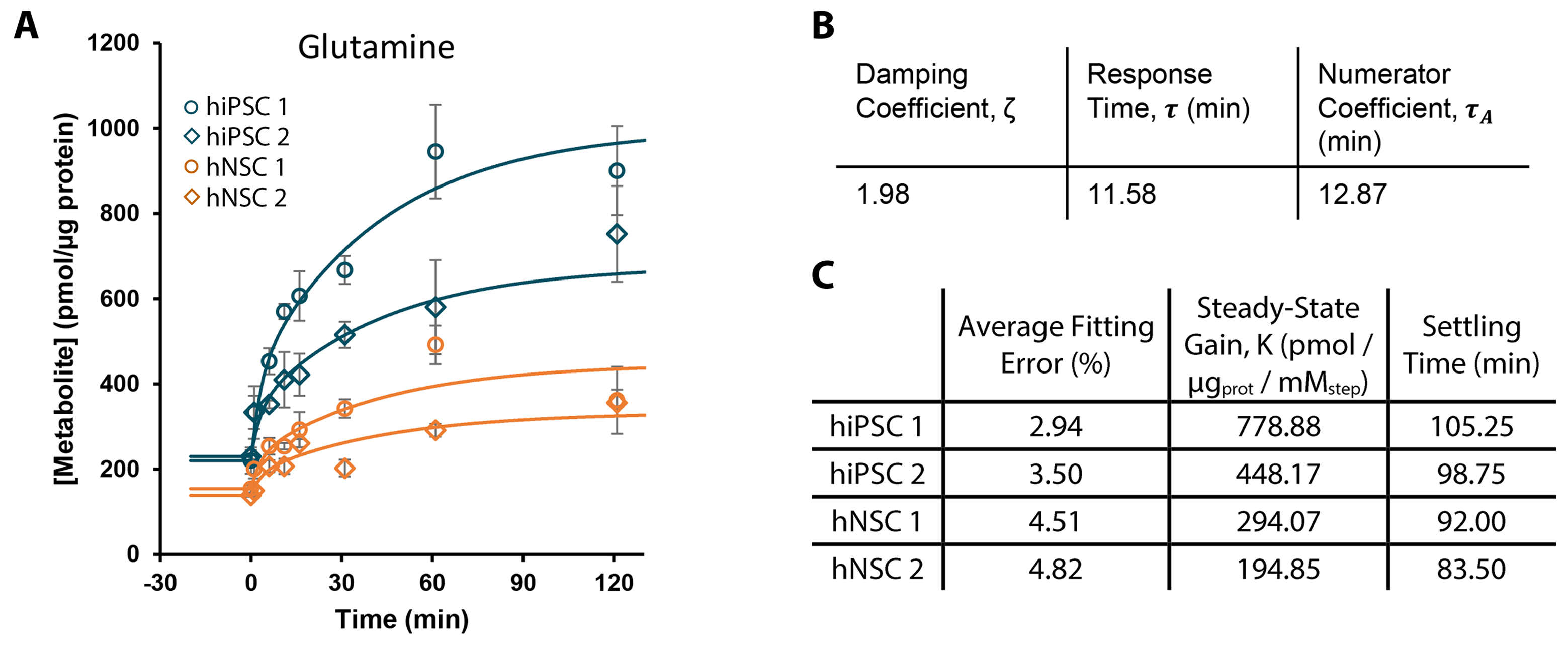

Supplement: S6 Fig — (A) Metabolic profile over two hours for each cell line. Experimental points: hiPSC 1—blue round circles, hiPSC 2—blue diamonds, hNSC 1—orange round circles and hNSC 2—orange diamonds. Simulated profiles: hiPSC in blue lines and hNSC in orange lines. Experimental data are represente as mean of sampling replicates and error bars represent standard deviation. (B) Parameters used for modeling glutamine profiles. (C) Step-response descriptors from glutamine profile modeling for each cell line. (TIF) [file pcbi.1007780.s006.tif]
