# Supplementary material for: Unveiling dynamic metabolic signatures in human induced pluripotent and neural stem cells
Source: PLoS Comput Biol. 2020 Apr 16;16(4):e1007780. doi: 10.1371/journal.pcbi.1007780 (PMC7188302; doi:10.1371/journal.pcbi.1007780)
Supplement: Attachment — Submitted filename: Reviewers_Comments.docx [file pcbi.1007780.s012.docx]

**Reviewer #1:** Vasconcelos e Sa et al. have applied process dynamics and process control models to metabolic data from human pluripotent (hiPSC) and neural stem cells (hNSC) following a step change in extracellular glutamine concentration. This is a very interesting study because new approaches are indeed needed to understand the dynamic and tightly regulated nature of metabolism. The authors’ strongest findings are that 1) metabolic control is cell-type specific, 2) metabolites such as histidine and alanine can exhibit no differences in initial concentration but different dynamics upon glutamine step change (Figs. 5 & 6), and 3) fold-change data can better discriminate hiPSC and hNSC than either initial or final concentrations (Fig. 5A). The metabolomic data appear to be of high quality, and the authors have used a very interesting stirred-tank bioreactor system that appears to maximize biological reproducibility.

That said, there are several issues which need to be addressed before this manuscript would be acceptable for publication in PLoS Comp Biol. Primarily, the authors have failed to demonstrate 1) that their approach has revealed “key metabolic regulators involved in cell fate decisions” and 2) that their approach has predictive value (as opposed to simply being a nonlinear regression that can’t predict out-of-sample data).

We would like to acknowledge the reviewer for the critical input and good suggestions, contributing to increase the quality of the present manuscript.

Major points:
1 - The authors should include their data in a public repository (eg, MetaboLights) or provide the data in Supplemental Tables. It is unacceptable that the authors state “The datasets used and/or analysed during the current study are available from the corresponding author on reasonable request” (page 21).

Following the reviewer’s suggestion, we have submitted the metabolomics data to the MetaboLights repository (accession number MTBLS1349), having modified the data availability statement accordingly (page 21/line 596). However, as the metabolic quantifications were outsourced to a service provider, we do not have access to all experimental data/details to complete and validate the MetaboLights submission. This is currently pending on an agreement between our provider (Biocrates) and the MetaboLights curators, which should be solved within the upcoming weeks. Also, the MetaboLights curators offered to provide any additional information on this process to the reviewers or editor upon request via email ([keeva@ebi.ac.uk](mailto:keeva@ebi.ac.uk)).

Nevertheless, in order to minimize this issue, we have also included an additional supplemental table (S2 Table), which contains the complete metabolic quantification data that was generated and analysed in this manuscript.

2 - The authors fit their process control model to one glutamine step change (2.5 to 15 mM), but they have not used their model to make any predictions about the response to other perturbations. For example, can a model fit to the 2.5 to 15 mM step change data successfully predict the dynamics upon switching to a different glutamine step change (i.e., 2.5 to 8.75 mM)? This would demonstrate that their models have some general applicability and would argue that this is a meaningful modeling approach.

We agree with the reviewer comments that this manuscript does not address predictive value of the proposed dynamic modelling approach. The goal of this first study was to (1) assess suitability of process control models to fit dynamic response of intracellular metabolic pools and (2) investigate untargeted dynamic analysis of the intrametabolome as a tool to find cell type-specific responses and potential targets for manipulation of human stem cell bioprocesses.

Model robustness and predictive testing would require a distinct experimental approach, focusing on specific metabolites/pathways (which would allow more samples over time and more accurate parameter determination), combined with varying perturbations (different metabolites, step intensities or schemes). Although this evaluation is critical, because it would require a separate approach, we believe it would be better addressed in a separate manuscript.

Note: A similar point was raised by Reviewer 2 in Comment 15.

3 - Figure 5A: The authors show that “fold-change of steady-states” is better at discriminating hiPSC and hNSC (using ROC) than either initial or final concentrations alone. This certainly argues that steady-state analysis is important. However, the authors have not shown that dynamic process control modeling improves the ability to discriminate between cell types. Data showing that transient profiles of metabolic pools (or the parameters derived from process control models) provides an even stronger discrimination of the cell types would significantly strengthen the authors’ conclusion that their process control modeling approach is valuable.

We value and agree with the reviewer’s suggestion. In Figure 5B, for instance for the metabolite tryptophan, the analysis by steady-state fold-changes would fail to discriminate between cell types (variance of the fold-change between replicates is high). However, the dynamic modelling shows that the tryptophan dynamics vary between cell types (Figure 5B and E). What could not be done in this article is to estimate precisely the 4 parameters in the process control equation (KM, τ_a_, τ and ζ – see equation 1 and 2) as that would require much more than 8 experimental points, which was the maximum experimentally possible number of samples. In these conditions of large degrees of freedom, different sets of estimated parameter values can be obtained while maintaining a similar profile. Therefore, the estimated parameters have a significant error of accuracy, despite the error of the graphic fit being low (as depicted in Figure 4) and doing statistical tests on these parameters (or consequential values such as settling time) would not be adequate. This would require larger volume experiments or parallel experiments to increase sampling frequency and number of samples.

4 - Figure 6C: the authors have identified metabolites with cell-specific dynamics (Fig. 6), but they have not provided any analysis that metabolites with cell-type specific dynamics are more important for cell fate or function than metabolites with non-specific dynamics. Such data would be essential for the authors’ conclusion that their approach has revealed “key metabolic regulators involved in cell fate decisions”. There is some discussion about amino acids and their important for hiPSC and hNSC (page 12), but there is no evidence here that amino acids and other cell-specific metabolites are **more important** than other non-cell-specific metabolites for stem cell phenotypes. The authors need to strengthen their analysis here or remove their substantially soften their claims that this approach reveals “key metabolic regulators”.

We agree with the reviewer’s comment that interpretation of the data should be more cautious in what it refers to the identification of metabolic regulators, so we substantially softened the claims made throughout the manuscript. The revised text has a clear separation between the achievements of this specific manuscript and the overall aim we would like to achieve in the future with this methodology.

We are currently studying the relevance of the metabolites that were identified as potential “key metabolic regulators” in this manuscript. However, given the complexity and timeline for the experiments required to prove their role in cell fate decisions, these will be included in a future manuscript.

The authors have changed the manuscript in the “Authors Summary” line 39 from “uncover key metabolic regulators” to “uncover potential key metabolic regulators”.

The following sentence in lines 316-319 (in initial submission) in the section of Results were deleted:

“Overall, the results presented this far gather together the several individual works on the influence of a specific amino acid in cell fate, and suggest that several amino acid pools are tightly regulated so that the homeostasis of cell phenotype is maintained.”

And replaced by the following sentence (line 320-322) that makes no claim that identified amino acids are key regulators of homeostasis:

“Overall, a large fraction of metabolites with cell type-specific dynamics, identified through this methodology, were amino acids.”

In the discussion section, we have removed the sentence 371-373 (in initial submission) because of the suggestion that decreasing steady-state values might be linked to intracellular homeostasis:

“This suggests that metabolites with cell type-specific dynamics may be important for maintaining the intracellular homeostasis after the sudden increase in glutamine pool levels.”

Note: A similar point was raised by Reviewer 2 in Comment 18

5 - The authors have provided no insight into what controls the cell-type specific differences. Is there gene expression or protein expression data that might explain why these cell-type specific differences occur?

The proposed study looking into gene expression and protein expression after the glutamine step increase would be very interesting. Specially to assess if the metabolic steady-state rearrangement was performed without changes in the protein population. After all, protein synthesis rate is usually in the time frame of hours [1,2] while some of the cell-type specific metabolites changed their levels in around 10 minutes. This reasoning has been added to the manuscript (lines 350-356). A future study, which could focus on specific metabolic pathways, should include transcript and protein samples to study at what level this regulation occurs.

Minor points:

6 - I was unable to access the Supplemental Tables. The link in the manuscript pdf took me to a word doc that described but did not actually contain the Supplemental Tables.

There could have been an error with the upload of Supplemental Tables, we will check during resubmission that all supplementary files are included.

7 - Figure 2: have the authors done any false discovery rate (FDR) correction? The figure says “Adjusted P=0.05” but I could not find any description of whether or how the authors did FDR correction of their p-values.

When we mentioned “adjusted P=0.05” it was meant to say that the value of 0.05 was converted to –log10 value, as was the data in the charts. We realize this was a poor word choice and have corrected it in the resubmitted manuscript. FDR was not performed as the goal of Fig2A was to give an initial indication of what metabolic pathways had increased or decreased metabolic levels, rather than identify specific metabolites. FDR is commonly used for larger datasets [3], for instance for gene expression data where specific genetic changes are searched for.

8 - Figure 2: what time point is considered the “steady state”? It is not clear from the text or the legend what time point is used here. It is not clear how confident a reader should be in the authors’ assessment that these metabolites have reached steady state.

In preliminary experiments, we have profiled the dynamic response of some metabolites until 24 hours and observed that the intracellular concentration did not change after 2 hours. Therefore, the steady-state was considered to be reached at 2 hours. The data of this preliminary experiment is now included in a new supplemental figure (Fig S2) and the text was modified (line 134-135) to clarify this rationale. Moreover, Fig 5B also provides a more visual demonstration of the time needed for some amino acids to reach their steady-state.

9 - The authors state that their model “successfully fitted at least 60% of the metabolites for each cell line” (page 8), but they have provided no discussion about why the model works for some but not other metabolites. Do the authors have any insight into why this modeling approach succeeds or fails for a given metabolite? Is there some shared characteristic among the fitted and not-fitted metabolites?

This is a very interesting question, but unfortunately one that we cannot answer based on this first study. As mentioned in that paragraph (line 217-220), the chosen model for fitting was the simplest one that could incorporate inertia in response. In order to get 100% of successful fittings, more complex models (and different types) would have to be used. That would allow us to detect different groups of dynamic response but would require more sampling points, as the number of parameters to be fitted would increase. After all, the network dynamics are not equivalent for every metabolic node (for instance glucose 6-phosphate versus a more central pyruvate). Increasing the sampling points would not be feasible within the initial 30 minutes after the glutamine step (where most of the variation is observed), as we have already used the minimum sampling time interval experimentally possible.

10 - Figure 4: it is unclear what part of this figure demonstrates the “mathematical modeling”. Is it the solid line in the figure? Probably, but the authors have not clearly stated that the solid line comes from their process control models.

We have modified the figure 4 caption according to the reviewer’s comment: “Solid lines represent the mathematical model fitted to the experimental data.” (lines 238-239).

11 - Figure 6: Are there classes / pathways of metabolites enriched in the identified metabolites with cell-specific or non-specific dynamics? The authors claim that the identification of amino acids is significant, but there is no statistical justification for this observation. The authors should attempt metabolite enrichment analysis or similar between these two classes of metabolites.

Following the reviewer’s suggestion, the authors have attempted to perform an enrichment analysis through the software MetaboAnalyst. However, the metabolic data set, after extracting cell type-specific and non-specific metabolic dynamics from the model, is not comparable in size with a typical high-throughput data set such as transcriptomics or proteomics used for pathway analysis [4], meaning that the obtained analysis comes with few statistical significant enriched pathways. In order to perform an accurate pathway analysis, the experiment would require a much larger number of metabolic methods so that the fraction of metabolites with conserved dynamics could perhaps be in range of hundreds.

12 - The data for alanine and histidine is shown twice: Fig. 5B and Fig. 6D.

We have now merged Figures 5 and 6 thus avoiding this repetition.

Reviewer #2**:** The authors of this manuscript describe metabolic responses of two human stem cell lines to step changes in glutamate availability. This dynamic, short-term response analysis of metabolite concentration is fairly novel and provides interesting insights into dynamics of metabolite pools. Furthermore, the use of control theory to model metabolite dynamics is new and potentially enlightening. Overall, the experiment is well-designed and metabolite analysis is also a strength. The manuscript is well-written and easy to follow. The simplicity of the study has both advantages and drawbacks. On one hand it provides a clear proof-of-concept, but it also leaves the reader with many unanswered questions. This work would be a valuable addition to stem cell metabolism literature as a proof-of-concept of the authors’ novel approach, but I encourage the authors to think about how additional analysis of system robustness and dynamics can be added to this study. Also, indications of how future studies will be designed would be valuable.

We thank the reviewer for valuable criticism and suggestions to improve this manuscript.

Specific comments:

13. My most significant comment involves a lack of pathway level analysis. While investigating individual pathways is useful, some analysis and discussion of how metabolites in the same pathway change (e.g. energetic and amino acid synthesis pathways) would go a long way to convincing the reader that this methodology can identify network dynamics accurately.

Following the reviewer’s suggestion, the authors have attempted to perform an enrichment analysis through the software MetaboAnalyst. However, the metabolic data set, after determining metabolites with a statistically significant change in their steady-state, is not comparable in size with a typical high-throughput data set such as transcriptomics or proteomics used for pathway analysis [4], meaning that the obtained analysis comes with few statistical significant enriched pathways. In order to perform pathway analysis, the experiment would require a much larger number of metabolic methods so that the fraction of metabolites with steady-state changes could perhaps be in range of hundreds.

Nevertheless, the volcano plots and the Pearson’s correlation coefficients in Figure 2 suggest what are the common pathways that change either in hiPSC or in hNSC, independently of the cell line.

14. p. 5, last sentence. It would be useful to show data demonstrating the system reached a steady state by 2 hr in a supplemental figure.

In preliminary experiments, we have profiled the dynamic response of some metabolites until 24 hours and observed that the metabolic intracellular concentration did not change after 2 hours. Therefore, the steady-state was considered to be reached at 2 hours. Indeed, the calculated settling time after modelling the dynamic metabolic profiles show that almost all metabolites reach the new steady-state before 2 hours after the glutamine step (see S4 Table). Fig.5B also provides a more visual demonstration of the time needed for some amino acids to reach their steady-state.

Following the reviewer’s suggestion, we have added a new supplemental figure (S2 Fig), showing the intracellular concentration of several amino acids until 24 hours after the glutamine step.

15. System robustness should be discussed and investigated, at least in a modeling approach. For example, is the system reversible and how does it respond to step changes of different magnitude, or ramped changes (ie fed batch reactor). This analysis could be done for a subset of the metabolites and pathways of interest in the authors’ experimental data.

We agree with the reviewer’s comments that this manuscript does not address predictive value of the proposed dynamic modelling approach. The goal of this first study was to (1) assess suitability of process control models to fit dynamic response of intracellular metabolic pools and (2) investigate untargeted dynamic analysis of the intrametabolome as a tool to find cell type-specific responses and potential targets for manipulation of human stem cell bioprocesses.

Model robustness and predictive testing would require a distinct experimental approach, focusing on specific metabolites/pathways (which would allow more samples over time and more accurate parameter determination), combined with varying perturbations (different metabolites, step intensities or schemes). Although this evaluation is critical, because it would require a separate approach, we believe it would be better addressed in a separate manuscript.

16. Overall the statistical analysis appears strong. I have one concern (p. 6, line 155). A t-test comparing two metabolites at 5% significance will determine whether 2 metabolites concentrations are different with 95% confidence. If I understand the test correctly, it’s not appropriate to use this to say two metabolites are not the same. The authors should clarify their test or perform a more appropriate test.

The referred t-test is used to build the volcano plots on Fig 2A, where each data point is a metabolite. This t-test compares two steady-states, one before the glutamine step increase and another one after 2 hours, and not for comparing two metabolites. We have modified the text to improve clarity (lines 157-163):

“For each metabolite, changes in steady-states were determined by calculating the ratio of final to initial average molar quantities per protein (fold-change). In order to identify statistically significant changes in steady-states, steady-state values, before the initial glutamine step increase and 2 hours after, were statistically compared by a two-sample t-test at 5% significance level. If null hypothesis prevailed, the steady-states before and after glutamine step were considered to be the same.”

17. A discussion and/or data of how metabolic networks change would be helpful. Responses are quite rapid. How much of the response is the result of new enzyme synthesis vs. pathway flux changes utilizing existing enzymes?

The proposed study looking into gene expression and protein expression after the glutamine step increase would be very interesting. Specially to assess if the metabolic steady-state rearrangement was performed without changes in the protein population. After all, protein synthesis rate is usually in the time frame of hours [1,2] while some of the cell-type specific metabolites changed their levels in around 10 minutes. A future study, which could focus on specific metabolic pathways, should include transcript and protein samples to study at what level this regulation occurs.

18. Line 318-319. “…suggest that several amino acid pools are tightly regulated so that the homeostasis of cell phenotype is maintained.” This is an interesting idea but lacks support. It isn’t clear that the cells can be maintained long term after switching the metabolic environment. Also, more importantly, it isn’t clear that the amino acid pools are necessary to maintain cell state. I suggest moving this point to the discussion, and being more clear this is speculation.

We agree with the reviewer’s comment that interpretation of the data should be more cautious in what it refers to the identification of metabolic regulators and substantially softened the claims made throughout the manuscript. The revised text has a clear separation between the achievements of this specific manuscript and the overall aim we would like to achieve in the future with this methodology.

We are currently studying the relevance of the metabolites that were identified as potential “key metabolic regulators” in this manuscript. However, given the complexity and timeline for the experiments required to prove their role in cell fate decisions, these will be included in a future manuscript.

The authors have changed the manuscript in the “Authors Summary” line 39 from “uncover key metabolic regulators” to “uncover potential key metabolic regulators”.

The following sentence in lines 316-319 (in initial submission) in the section of Results were deleted:

“Overall, the results presented this far gather together the several individual works on the influence of a specific amino acid in cell fate, and suggest that several amino acid pools are tightly regulated so that the homeostasis of cell phenotype is maintained.”

And replaced by the following sentence (line 320-322) that makes no claim that identified amino acids are key regulators of homeostasis:

“Overall, a large fraction of metabolites with cell type-specific dynamics, identified through this methodology, were amino acids.”

In the discussion section, we have removed the sentence 371-373 (in initial submission) because of the suggestion that decreasing steady-state values might be linked to intracellular homeostasis:

“This suggests that metabolites with cell type-specific dynamics may be important for maintaining the intracellular homeostasis after the sudden increase in glutamine pool levels.”

Regarding the question if cells can be maintained for long after switching the metabolic environment, some ongoing experiments performed in hiPSC as a follow-up work outside of this manuscript’s scope, suggest that pluripotency markers are maintained 72 hours after a glutamine step increase.

**Reviewer #3:** The authors describe broad metabolic changes in response to short-term glutamine exposure in human iPSC and NSC, identifying changes in both steady state and dynamic (within 30 minutes) metabolic subgroups. In addition, they describe cell-type specific changes. It is well known that metabolic change occurs rapidly (long before changes in gene expression etc), so it is not surprising that upon glutamine challenge cells compensate by immediately modulating amino acid utilisation. Equally, it is not surprising that different cell types respond to the same challenge in a different manner, given that their baseline metabolic profile is different, and therefore have different metabolic needs. The physiological relevance of the challenge used is not clear, and overall the manuscript lacks sufficient context for the biological researcher to meaningfully interpret. This is significant given that the authors rationale for this work (abstract) being to contribute to understanding what cells need (as opposed to how they respond) given that media formulations are essentially based on tissue culture designed in the 1950s.

The manuscript requires editing by an English speaker as there are numerous errors.

We thank the reviewer for the comments that contributed to improve the quality of the manuscript. We will address specific concerns bellow.

Specific comments:

19. The authors state that metabolic changes are considered as ‘generally consistent’, thereby suggesting cell-type specific changes (although they do denote this as ‘cell-specific’ which is confusing). Based on their data, it is quite apparent that each cell line displays a vastly different response to the challenge (within cell-type). This is then made particularly apparent on line 218 where 99, 134, 116 and 71 metabolies were fitted to a mean fitting area of which only 60% were successful, as well as in Figure 6A where only 50% of metabolites are shared between hiPSC lines (although which metabolites this represents is not clear), and even fewer between the 2 NSC lines. As a result, the conclusions made are questionable, particularly those around preferred metabolic pathways (and the section on fitting metabolic profiles).

As suggested by the reviewer and to improve precision and clarity, all mentions of “cell-specific” were changed to “cell type-specific”.

The reasons for 60% of metabolites being considered as successfully fitted to the mathematical model were discussed in the reply to Reviewer 1, Comment 9 and the respective clarification was added to the manuscript.

The cell type-specific changes are evidenced in Fig. 5C, where we demonstrate that for many metabolites the model fits the two hiPSC lines or the two hNSC lines with the same parameters, with a mean fitting error below 4%. Moreover, when defining the minimal mean fitting error in Fig. S3, it can be seen that the intersection of hiPSC lines and the intersection of hNSC lines offer the highest number of fits with shared parameters.

One of the underlying hypothesis of this work was that some metabolites have dynamics that are more controlled in comparison with other metabolites. So, it was expected that some metabolites have more “degrees of freedom” to respond while other metabolites respond in some characteristic manner. The principle here is similar to the classical study where metabolic pools are compared between two different cell lines of the same cell type, and the metabolites with equivalent concentrations are considered biomarkers of that cell type (example in [5] where multiple cell lines were used to eliminate false positives).

20. While the data would be of significant interest, no specifics are provided (i.e. details of actual metabolites changing beyond Figure 3 – which is steady-state, not the dynamics they suggest are key to understanding cell metabolism). For example, on line 373-5 of the discussion the authors note that ‘all metabolites with cell-specific dynamics and with decreased steady-state were amino acids. Some of the identified amino acids are known to have important cell regulatory functions…’. Yet no specific AAs are highlighted, which would be the key interest of those reading this paper. What metabolites common within a cell-type were fitted to their model (and in Figure 6A etc)? This makes it very difficult to reconcile with the discussion (suggesting that their method allowed an analysis of the essential and constant metabolic features of the cells), as well as the title (metabolic signatures) when no specific metabolites/signatures are divulged.

The sentence in line 387-388 in the discussion is trying to refer back to Figure 5E where it can be seen that “all metabolites with cell type-specific and with decreased steady-state were amino acids”. Figure 5C gives the number of metabolites with shared dynamics between hiPSC cell lines (25 metabolites) and between hNSC cell lines (5 metabolites) while Figure 5E lists them. Specifically, those are alanine, asparagine, glycine, histidine, leucine, methionine, proline, serine, threonine and tryptophan. In the last paragraph of Results section, examples of identified amino acids in cell regulatory functions are given.

21. The authors state that hNSC alter their metabolism in response to glutamine by the ‘upstream section of the TCA cycle’ (line 184), but this is difficult to discern for anything other than lysine, and lipids (not TCA).

We understand the reviewer’s comment because the evidence of increased metabolic pools in the upstream section of the TCA cycle is indirect. In contrast to hiPSC, the lipidic pools of phosphatidylcholines and sphingomyelins are increased in hNSC and the metabolic pathway for that lipid synthesis usually must pass through citrate. This citrate either is generated from oxaloacetate / pyruvate or from reductive carboxylation by the conversion of alpha-ketoglurate to citrate. Indeed, the metabolites that would be involved to fuel the former type of conversion into citrate for lipid synthesis - fumarate, malate, oxaloacetate – had very low increases or even have decreased (red bar) their intracellular concentration. Moreover, as mentioned later in the paragraph, that hypothesis is corroborated by previous studies using metabolic flux analysis.

Nevertheless, we altered the lines 190-193 to:

“On the contrary, in hNSC, the glutamine shock had no effect on increasing the metabolites in the downstream section of the TCA cycle. Instead, the number of lipidic pools for which the steady-state increased was much higher in hNSC than in hiPSC, suggesting an increase in metabolic flux through the upstream section of the TCA cycle.”

22. If a steady-state metabolic profile is reached within a short period of time, how do these profiles compare with previously reported profiles for each cell type? The authors state (line 365) that their results closely match data obtained by carbon-labelling. If these analyses have been performed, they should be included within the manuscript.

We added the references of published works in lines 374-377:

“Interestingly, our results closely match the data obtained by carbon-labelling experiments, usually more expensive and labor-intensive [9,22].”

23. The use of the term ‘pluripotent’ implies both embryonic and induced pluripotent stem cells, however the authors have only analysed the latter, which are metabolically very different (as referenced in the paper – Panopoulos et al) and have also been documented to modulate metabolism in response to metabolite availability with varying abilities (which may relate to different methods of generation; I note the two lines used were generated either by retroviral or episomal reprogramming in the current submission). They should therefore not generalise to PSC throughout the manuscript (or in the title) and additional analyses should be performed to include examination of hESC. It would also be useful for the authors to comment within the manuscript about the differences between the cell lines, particularly in relation to differences in source/generation etc. What is the culture history (media used) for these lines?

We agree with the reviewer’s comment on the functional and metabolic differences between iPSC and ESC and modified the manuscript title accordingly:

“Unveiling dynamic metabolic signatures in human induced pluripotent and neural stem cells”

In all other references to the pluripotent stem cells used in this work, the authors have checked if these were always preceded by the term “induced” or by the acronym “hi”.

The goal of the present manuscript was to provide a proof-of-concept that the methodology could be used to probe dynamic metabolic responses, and not to establish metabolic distinctions between multiple cell origins/types. Nevertheless, we agree with reviewer on the importance of clarifying the background history of the cell lines used. This is available in public repositories, and we have added the corresponding Research Resource Identifiers (RRIDs) in the Materials and Methods section to allow easy access to relevant information:

“Primed hiPSC IMR90-4 (RRID: CVCL_C437) were purchased from WiCell and WTC-11 (RRID: CVCL_Y803) were obtained from the J. David Gladstone Institutes, designated throughout the text by hiPSC 1 and hiPSC 2, respectively”

“hNSC 2 was derived from hiPSC line RIi001-A (RRID: CVCL_C888), as previously described [38], and designated throughout the text as hNSC 2.”

24. What is the physiological relevance of using glutamine (and at the concentration specified) to challenge either cell type? Likewise, what, if anything, does the challenge do functionally to these cells? The authors could have assessed cell viability after the challenge (there is potential that cell death or differentiation is induced in the short window of time being examined, which may contribute to different metabolic changes seen between cell types), but more importantly, the authors should include measures of pluripotency/potency and differentiation capacity, as well as gene expression and epigenetics. Similarly, are cells still proliferating equivalently before and after the challenge? Without these latter analyses the authors can not conclude that their method is suitable for identifying the most effective phnotype modulator (line 387) the ‘identification of metabolites characteristics of a cell phenotype’ or ‘metabolic signatures of stemness’ (line 394).

The goal of this work was to demonstrate that the dynamic responses between hiPSC and hNSC are different, at least for some metabolites. For that, glutamine was chosen because this metabolite is consumed by hiPSC [6] and by hNSC [7–9]. By using the same metabolite for the challenge, it is possible to compare dynamic responses between cell types per metabolite. The level of step increase of glutamine was chosen, to facilitate data analysis. With metabolic responses covering a higher range of concentration (seen in variations of steady-state changes and in oscillations), the dynamic profile is fitted with higher confidence.

Regarding the consequences of the glutamine challenge to the functionality of the cells, this does not affect the dynamic response observed in each cell type. Nevertheless, in ongoing experiments performed for subsequent work, the phenotypic markers and cell viability of hiPSC remained unchanged for 72 hours after the glutamine challenge.

We agree with the reviewer’s comment concerning the claim that this method is not proven for identifying phenotypic modulators and have revised the full manuscript accordingly. For a more detailed list of changes please refer to Reviewer 1, Comment 4.

Minor comments:

25. Line 54: in place of ‘in this last work’ simply state ‘Pavlova et al 2018’.

Following the reviewer’s suggestion, we altered that sentence in line 54 for:

“For instance, it was found that asparagine uptake in mammalian cells preferentially occurs in a glutamine-deficient environment and does not occur when glutamine is present [7].”

26. It is unclear how many biological replicates were performed, or whether the same stirred bioreactor culture was merely sampled on different days (i.e. a single biological replicate).

The financial and time investment in these bioreactor experiments and consequent metabolic analysis are significant. We considered more important to have replicates of the same cell type with different cell lines than replicates of bioreactors with the same cell line. Thus, two bioreactors (and cell lines) were used to study hNSC metabolism and two to study hiPSC metabolism.

27. Please detail the ammonium accumulation analyses in the methods and results.

Following the reviewer’s suggestion, we have added the ammonia accumulation results in Supplemental Figure S1, having also modified the Materials and Methods section accordingly.

In the Results section we added a remark on the ammonia concentration in lines 132-134:

“Furthermore, the quantity of glutamine added did not alter significantly the osmolarity or the ammonia concentration (Fig S1) or the concentration of glucose and lactate in bioreactor culture media (data not shown).”

28. It is unclear whether excluded data points (lines 143-152) relate to metabolites or samples as a whole. If they relate to samples, then this particular sample should be removed from all analyses (not just certain metabolites where they are perceived as outliers).

Two types of pre-processing steps were performed and described in lines 142-156: one concerning samples and other concerning metabolites.

For each cell line, there were 8 time-points with 3 sampling replicates each (24 samples per cell line). Samples whose values of metabolite concentrations, for all 201 metabolites, were systematically far from the values of the two other replicates at the same time-point, were removed. The rationale here is that some error must have been introduced during sample processing, causing a systematic deviation of metabolite concentrations (e.g., loss of protein pellet, loss of supernatant containing the metabolites, inefficient intracellular metabolite extraction). So, from the 24 samples per cell line 2 samples in hiPSC 1, 2 samples in hiPSC 2, 1 sample in hNSC 1, and 2 samples in hNSC 2 were considered outliers. This is in accordance with the reviewer’s suggestion.

The second pre-processing relates to metabolites, which were removed from the dataset when the majority of their concentrations (8 time-points with averaged concentration) had null values or below the limit of detection (LOD) or had a relative standard deviation that implicated that the calculated average had low statistical confidence. Possible reasons for these outliers are: 1) intracellular concentration of certain metabolites is low, being below or close to the limit of quantification, and 2) variations of extraction efficiency depending on metabolic class. This step too was applied to all occurrences of a metabolite, not to specific metabolites within specific samples.

To make it clearer that two pre-processing data operations were performed, we added the following statement to line 144:

“Two types of pre-processing operations were performed: (i) at sample level, for removing samples that were considered as mistreated during sample preparation/processing (i.e., presenting metabolite concentrations systematically far from its equivalent replicates) and (ii) at metabolite level, for removing metabolites that could not be quantified with accuracy or at all (i.e., presenting null values or below the limit of detection).”

29. Viability analysis should be quantified (%). The PI staining in Figure 1 is very difficult to visualise.

This work was performed using a well-established and validated system for culturing hiPSC and hNSC as spheroids. Previous reports have demonstrated the validity of such system to maintain highly viable cells cultures [10–12]. Also, as observed by the fluorescence images (Fig 1A), the proportion of PI-positive is very low. Attempting to count the number of PI- and FDA-positive cells would result in a very inaccurate measurement, given the thickness of spheroids that produces high out-of-focus light rendering it extremely difficult to confidently discriminate individual cells on non-confocal imaging.

30. Figure 2: do the points on the graph represent samples (as a whole) or specific metabolites?

Data points in Figure 2A and 2B are specific metabolites. We have added a clarification to Figure 2 caption accordingly.

31. Line 236: Please expand on the specific transient dynamics that may contribute to distinct conserved metabolic features.

The message of the sentence and of Fig 5B was that profiling the transient adaptation of certain metabolites after the glutamine step is a better metabolic characterization tool than looking solely to steady-states concentrations. Even when considering the more sophisticated tool of fold-changes of steady-states, it is entirely possible that the same metabolite, in both cell types, can have the same fold-change but in one cell type show oscillations or overshoots while in the other cell type that behaviour is not observed. Therefore, the ability of distinction between cell types is increased when profiling transient dynamics. This was clarified in the text in lines 250-255.

32. The authors need to cite the relevant stem cell metabolic literature (rather than studies on E.coli etc). Please also specify the cell types examined in cited literature (e.g. line 350-351 ‘previous works sharing related concepts or objectives focus on glycolysis and the PPP’ which does not relate to the cell types examined in the current study).

In that paragraph, we discuss the methodology for tracking the metabolic dynamics. The amount of work performed with dynamic metabolomics in human somatic cells is minimal, even more when considering human stem cells. For that reason, the authors have cited *E. coli* literature just for methodological considerations. Nevertheless, for observations regarding specific metabolites or pathways, the authors have tried to cite as much literature in human stem cell metabolism as possible – references [2], [3], [4], [6], [9], [13], [14], [15], [16], [21], [22], [25], [27] and [34].

Following the reviewer suggestion, we have specified the cell types examined in cited literature:

Line 114: “Indeed, this amino acid, a versatile donor of nitrogen and carbon atoms for diverse biosynthetic reactions, is preferentially consumed by proliferating mammalian cells in comparison with other amino acids [7].”

Line 116: “Nucleotides, non-essential amino acids and the anaplerotic substrates of the TCA cycle are all major biosynthetic products of glutamine in human cells [17].”

Line 130: “The absence of ammonia accumulation after the perturbation step (data not shown) corroborates that the final concentration of glutamine is not cytotoxic, as previously reported in murine PSC [21].”

Line 319: “In addition, serine as well as glycine, are involved in one-carbon metabolism that influence epigenetics in human cells [27].”

Line 360: “Previous works, in *E. coli* and human skin cells, sharing related concepts or objectives usually focused on glycolysis and PPP [29,31].”

Line 366: “Lipid levels have been found to be changed between hiPSC and hESC [34] and to promote important signaling for insulin-glucose homeostasis [35].”

Line 388: “Some of the identified amino acids are known to have important cell regulatory functions, for instance asparagine in protein synthesis in mammalian cells [7].”

33. Ref 34 is used to suggest differences between cell types, however this reference relates more specifically to differences in metabolic features between embryonic and induced pluripotent stem cells (i.e. both are ‘pluripotent’ and therefore considered the same cell type) rather than differences between iPSC and the somatic cells from which they were generated.

Following the reviewer’s suggestion, we added a clarification that the difference in metabolic features in line 365 is between hiPSC and hESC. The sentence was rewritten to:

“Lipid levels have been found to be changed between hiPSC and hESC [34] and to promote important signaling for insulin-glucose homeostasis [35].”

34. Line 364-365: references should be provided to support this statement.

We added references to support this statement (now in lines 374-376):

“Interestingly, our results closely match the data obtained by carbon-labelling experiments, usually more expensive and labor-intensive [9,22].”

35. Line 415: Please include the passage numbers assessed and the incubator conditions used. Also, why were cells supplemented with Y-27632 when this is known to alter metabolism?

Passage numbers used for the different cells are now specified in the Materials and Methods section (lines 442-443):

“hiPSC 1, hiPSC 2, hNSC 1 and hNSC 2 were used for these experiments at cell passage number P40, P36, P12 and P34, respectively.”

Supplementation of human stem cell cultures with Y-27632, a ROCK pathway inhibitor, is standard in the field in order to maintain cell viability upon cell passaging. These cells require constant cell contact stimuli, otherwise programmed cell death is triggered. Therefore, Y-27632 was added to the culture medium only at inoculation of either culture plates or bioreactors, to overcome this effect. For medium exchange along culture time, this supplement was no longer added. As, the metabolic experiments were only performed 2-3 days after cell inoculation is reasonable to assume that the effect of ROCK inhibitor on the metabolism is minimal. Moreover, analysis of hiPSC aggregates show that their phenotypic markers are maintained intact (Fig 1B).

To make this clearer and to expand on the incubator conditions the Materials and Methods section was altered accordingly:

“At cell passage, mTeSR1™ was also supplemented with 5 µM of ROCK inhibitor Y-27632 (Calbiochem). Complete medium exchange was performed every day. Cells were maintained under humidified atmosphere with 5 % CO2, at 37 °C.”

“hNSC were maintained under humidified atmosphere with 5 % CO2 and 3 % O2, at 37 °C.”

36. Please provide further details of the ‘Energy Metabolism Assay’ as this product no longer seems to be available (or is not produced by Biocrates).

We have submitted to MetaboLights database (accession number: MTBLS1349) all the available details on the Biocrates assays used in this manuscript. Nevertheless, the validation of this submission is still pending on an agreement between our provider (Biocrates) and the MetaboLights curators, as detailed in the reply to Reviewer 1, Comment 1. Please note that a substantial part of the experimental procedures is proprietary of the company and therefore were not disclosed.

Information on the Energy Metabolism Assay can also be found in the following webpage: <https://biocrates.com/images/Metabolic-Phenotyping-Folder_Web_2018.pdf>. In this assay, Biocrates has provided us data on 13 metabolites: glucose, ribose, 2-hydroxyglutaric acid, alpha-ketoglutaric acid, fumaric acid, malic acid, succinic acid, 2-hydroxybutyric acid, 3-hydroxybutyric, acid, lactic acid, sum of pyruvate and oxalacetic acid, citric acid, isocitric acid (usually 8 amino acids are also included in this kit but these have been analysed in another Biocrates’ kit).

37. It is unclear how much ‘randomness’ (i.e. variability) contributed to quantification of metabolites, and I am concerned as to why data were subsequently normalised. What is the precedence for using the normalisation equation?

Volumetric sampling of spheroid cultures tends to be more susceptible to biomass variation in comparison to single cell suspension cultures, which is easily explained as spheroid density (10^2^-10^3^ spheroid/mL) is usually lower than typical densities of single cell cultures (10^5^-10^6^ cell/mL). Therefore, repeated samplings might result in slightly different cell mass. To model intracellular dynamic profiles, it is important that the different time-points are analysed for equivalent cell mass bases, which requires normalization of metabolomics data [13]. Thus, protein mass, given that cell protein content can be considered reasonably constant, was quantified for each sample and this was used to normalize metabolite concentration so that the new units were mol/μg cell protein.

38. The authors should provide a list of all (201) interrogated metabolites. Particularly with regard to 'energy metabolism' denoted in green in Figure 2.

Following the reviewers’ suggestions, the metabolomics dataset will be available in a public repository (MetaboLights, accession number MTBLS1349), including the identity of the 201 metabolites. We have also included an additional supplemental table (S2 Table), which contains the complete list of metabolites interrogated in this manuscript.

39. In Figure 3, do the authors mean production and consumption for 'positive fold change' and 'negative fold change' respectively?

Correct, positive fold change was used in the text to mean increased intracellular metabolic concentration, and the negative fold change decreased intracellular concentration. Clarification was added to the caption of Figure 2:

“Positive fold-change means increase of intracellular metabolic pool level after glutamine step.”

40. Figure 4: the authors use alanine as an example of a metabolite which maintains the same steady-state, yet it appears to shift quite dramatically in iPSC. This needs to be clarified.

Despite intracellular concentration of alanine being highly affected immediately after the glutamine step (during the initial 30 minutes), the final steady-state (after 2 hours) estimated by the mathematical model has no significant difference from initial intracellular concentration.

41. Figure 6: Please define what the numbers in brackets (e.g. hiPSC 1 Set (37)) indicate. It is difficult to understand what 6B is depicting.

The numbers set in brackets represent the number of metabolites with fitted dynamics for each cell line. We have modified the figure caption accordingly.

**Have all data underlying the figures and results presented in the manuscript been provided?**
Large-scale datasets should be made available via a public repository as described in the *PLOS Computational Biology* [data availability policy](http://journals.plos.org/ploscompbiol/s/data-availability), and numerical data that underlies graphs or summary statistics should be provided in spreadsheet form as supporting information.

Reviewer #1: No: The authors should include their data in a public repository (eg, MetaboLights) or provide the data in Supplemental Tables. It is unacceptable that the authors state “The datasets used and/or analysed during the current study are available from the corresponding author on reasonable request” (page 21).

Reviewer #2: None

Reviewer #3: Yes

References:

1.	Kristensen AR, Gsponer J, Foster LJ. Protein synthesis rate is the predominant regulator of protein expression during differentiation. Mol Syst Biol. Nature Publishing Group; 2013;9: 1–12. doi:10.1038/msb.2013.47

2.	Bohé J, Aili Low JF, Wolfe RR, Rennie MJ. Latency and duration of stimulation of human muscle protein synthesis during continuous infusion of amino acids. J Physiol. 2001;532: 575–579. doi:10.1111/j.1469-7793.2001.0575f.x

3.	Chong EY, Huang Y, Wu H, Ghasemzadeh N, Uppal K, Quyyumi AA, et al. Local false discovery rate estimation using feature reliability in LC/MS metabolomics data. Sci Rep. Nature Publishing Group; 2015;5: 1–11. doi:10.1038/srep17221

4.	García-Campos MA, Espinal-Enríquez J, Hernández-Lemus E. Pathway analysis: State of the art. Front Physiol. 2015;6: 1–16. doi:10.3389/fphys.2015.00383

5.	Sperber H, Mathieu J, Wang Y, Ferreccio A, Hesson J, Xu Z, et al. The metabolome regulates the epigenetic landscape during naive-to-primed human embryonic stem cell transition. Nat Cell Biol. 2015;17: 1523–35. doi:10.1038/ncb3264

6.	Tohyama S, Hattori F, Sano M, Hishiki T, Nagahata Y, Matsuura T, et al. Distinct metabolic flow enables large-scale purification of mouse and human pluripotent stem cell-derived cardiomyocytes. Cell Stem Cell. Elsevier Inc.; 2013;12: 127–37. doi:10.1016/j.stem.2012.09.013

7.	Yeo H, Lyssiotis C a, Zhang Y, Ying H, Asara JM, Cantley LC, et al. FoxO3 coordinates metabolic pathways to maintain redox balance in neural stem cells. EMBO J. Nature Publishing Group; 2013;32: 2589–602. doi:10.1038/emboj.2013.186

8.	Palm T, Bolognin S, Meiser J, Nickels S, Träger C, Meilenbrock R-L, et al. Rapid and robust generation of long-term self-renewing human neural stem cells with the ability to generate mature astroglia. Sci Rep. Nature Publishing Group; 2015;5: 16321. doi:10.1038/srep16321

9.	Kleiderman SM, Sá J V, Teixeira AP, Brito C, Gutbier S, Evje LG, et al. Functional and phenotypic differences of pure populations of stem cell-derived astrocytes and neuronal precursor cells. Glia. 2015;64: 1–21. doi:http://hdl.handle.net/10993/22515

10.	Simão D, Silva MM, Terrasso AP, Arez F, Sousa MFQ, Mehrjardi NZ, et al. Recapitulation of Human Neural Microenvironment Signatures in iPSC-Derived NPC 3D Differentiation. Stem Cell Reports. 2018;11: 1–13. doi:10.1016/j.stemcr.2018.06.020

11.	Simão D, Arez F, Terasso AP, Pinto C, Sousa MFQ, Brito C, et al. Perfusion Stirred-Tank Bioreactors for 3D Differentiation of Human Neural Stem Cells. In: Turksen E, editor. Bioreactors in Stem Cell Biology. New York, NY: Humana Press; 2016. pp. 129–42. doi:10.1007/7651

12.	Abecasis B, Aguiar T, Arnault É, Costa R, Gomes-Alves P, Aspegren A, et al. Expansion of 3D human induced pluripotent stem cell aggregates in bioreactors : Bioprocess intensification and scaling-up approaches. J Biotechnol. Elsevier B.V.; 2017;246: 81–93.

13.	Gowda H, Ivanisevic J, Johnson CH, Kurczy ME, Benton HP, Rinehart D, et al. Interactive XCMS online: Simplifying advanced metabolomic data processing and subsequent statistical analyses. Anal Chem. 2014;86: 6931–6939. doi:10.1021/ac500734c
