# Supplementary material for: Unveiling dynamic metabolic signatures in human induced pluripotent and neural stem cells
Source: PLoS Comput Biol. 2020 Apr 16;16(4):e1007780. doi: 10.1371/journal.pcbi.1007780 (PMC7188302; doi:10.1371/journal.pcbi.1007780)
Supplement: Attachment — Submitted filename: Reviewers_Comments_2020.02.13.docx [file pcbi.1007780.s013.docx]

**Reviewer #1:** In this revised manuscript, Vasconcelos e Sa et al. have 1) improved availability of their metabolomic data; 2) softened claims that their approach has discovered “key regulators” of cell fate decision; 3) justified their choice of 2 h as steady state for this system.

The value of this manuscript still lies in the demonstration that fold-change of steady state metabolite concentrations are 1) cell-type specific (Fig. 2B) and 2) improve the ability to discriminate cell types (Fig. 5A). This data presents a valuable contrast to recent high-profile publications of steady state metabolite concentrations across 100’s of cell lines (e.g., PMID 31068703). I do, however, wish the authors had more thoroughly demonstrated either 1) that dynamic process control modeling improves the ability to discriminate between cell types or 2) that metabolites with cell-type specific dynamics are more important for cell fate or function than metabolites with non-specific dynamics.

We would like to acknowledge the reviewer again for the critical input, contributing to increase the quality of this manuscript. The last two points raised by the reviewer are indeed our motivation for the new approach introduced in this manuscript, but this first attempt and dataset did not allow us to address them yet. We hope to be able to do so in subsequent work.

The authors are also thankful for mentioning that publication (PMID 31068703) and have cited it in the Discussion section, lines 366-367:

“in contrast with using intracellular metabolic levels for discriminating different cell types, as used recently in cancer cell lines [35].”

That said, I think the manuscript would be acceptable for publication pending correction of these minor points:

Minor points:
1 - The authors state in their response that false discovery rate correction “was not performed as the goal of Fig2A was to give an initial indication of what metabolic pathways had increased or decreased metabolic levels, rather than identify specific metabolites. FDR is commonly used for larger datasets [3], for instance for gene expression data where specific genetic changes are searched for.” The cited reference [3] in the Response (PMID 26596774) investigates an alternative method of FDR correction (“local false discovery rate (lfdr)) but does not suggest that metabolomics data does not require FDR correction. The authors should either apply this lfdr approach or else do traditional FDR correction (e.g., Benjamini-Hochberg) to Figure 2A. It’s simply wrong to ignore the FDR correction for this data (which is smaller than gene expression data but still large enough to warrant FDR correction).

Following the reviewers’ suggestion, the authors have applied the traditional FDR correction, by using the Benjamini-Hochberg algorithm, to the fold-change data. Figure 2A has been updated accordingly.

In Figure 2A, the authors have changed the legend to:

“(A) Volcano plots of steady-state changes of metabolites. The threshold for Type I error (α) is 0.05, with p-value corrected for multiple testing hypothesis, and the threshold for a relevant fold-change is 30% difference from initial steady-state. Positive fold-change means increase of intracellular metabolic pool level after glutamine step.”

In the Materials & Methods section “Data pre-processing and statistical analysis”, the following sentence was added:

“The Benjamini-Hochberg method was used to correct for multiple testing errors using a false discovery rate of 5% [44].”

In lines 164-167, faced with some decrease of statistical confidence in phosphatidylcholine fold-change’s alteration in hNSC 2, the authors have softened that observation by rephrasing the respective sentence:

“Volcano plots of steady-state changes indicate a decrease in the metabolic pools of aminoacids for both hiPSC and hNSC. These plots also suggest a trend towards an increase in central energy metabolic pools for hiPSC, while in hNSC lipids, mainly phosphatidylcholines, appear to be affected in their steady-state value (Fig 2A).”

2 - The discussion about the role of amino acids in cell fate decision that is currently in the Results section should be moved to the Discussion because this is speculation based on the authors’ results, rather than actual Results (Line 314: “After all, amino acids are known to be involved in cell fate decisions. For instance, a controlled level of intracellular methionine and of the enzymes involved in its metabolism has been shown to be crucial for maintenance of pluripotency in hiPSC [25]. In a specific subtype of breast cancer with stem-cell like properties, asparagine has been pointed out as a key factor for governing metastasis. Besides that, serine supports proliferation of the breast cancer cells [26]. In addition, serine as well as glycine, are involved in one-carbon metabolism that influence epigenetics in human cells [27].”)

We agree with the reviewer’s comment and have moved all the discussion about the role of amino acids in cell fate decision to Discussion. Lines 385-395:

“A controlled level of intracellular methionine and of the enzymes involved in its metabolism has been shown to be crucial for maintenance of pluripotency in hiPSC [30]. In a specific subtype of breast cancer with stem-cell like properties, asparagine has been pointed out as a key factor for governing metastasis [35]. Asparagine has also been shown as regulator of protein synthesis in mammalian cells [7]. Serine supports proliferation of the breast cancer cells [36]. In addition, serine as well as glycine, are involved in one-carbon metabolism that influence epigenetics in human cells [37]. Besides the biological importance of the identification of amino acids as having dynamics which are cell-type specific, this result suggests that targeted approaches for identification of amino acid dynamics may be sufficient for cell identity characterization, instead of the more complex and expensive untargeted metabolomics studies.”

Reviewer #2**:** While preliminary in nature, the approach here warrants publication. The authors plan to follow up on this publication in subsequent work to address the model robustness, which will be crucial toward establishing advantages offered by their control theory approach.

We thank again the reviewer for valuable criticism and suggestions to improve this manuscript. After the results of this initial study, the authors are designing new experimental and computational approaches that can address the model robustness and retrieve more precision on the values some potential enlightening dynamic parameters such as settling time and damping coefficient. The authors will also address the proposed study by the reviewer on uncovering what are the mechanisms that drive the metabolic adaptations.

**Reviewer #3:** The authors have adequately addressed the majority of concerns raised by all reviewers.

We thank the reviewer for the comments that contributed to improve the quality of the manuscript. We will address specific concerns bellow.

Specific comments:

3 – If phenotypic markers and cell viability have been assessed, it would be useful to include a note to this effect within the manuscript. (Continuation of point 24 in the first reply to reviewers’comments.)

The authors agree with the importance of clarifying that this methodology does not affect cell phenotype. In ongoing experiments for subsequent work, focusing on the phenotypic impact of metabolic challenges, we have assessed the expression of pluripotency markers in 2D hiPSC cultures. These analysis have shown no significant modulation on the expression levels of such markers, up to 72 hours after the glutamine challenge.

Following the reviewer’s suggestion, we have added the following statement in line 135:

“Moreover, cell phenotype does not seem to change after glutamine perturbation: pluripotency markers and cell viability of 2D hiPSC cultures have remained unchanged for 72 hours after glutamine perturbation in subsequent experiments.”

4 – How replicates were performed needs to be included in the manuscript. While it is appreciated that multiple stirred reactors would not be available, it needs to be clarified whether the replicates represent different thaws of the same cell line repeated (or just a single thaw resampled on different days) as it remains unclear. If the latter then this would not be a replicate.

There were two types of replicates in this study: 1) cell type replicates meaning two different cell lines of hiPSC and two different cell lines of hNSC; and 2) sampling replicates at each time-point in one bioreactor done for each cell line (e.g. three samples at 10 min after glutamine step in the bioreactor of IMR90-4 cell line of hiPSC). The authors considered that the use of different cell lines as biological replicates, could then incorporate greater biological variability into the study than using multiple thaws or passage number of the same cell line. In line 76 we mention the use of four cell lines for testing the study hypothesis: “In order to test our hypothesis, two human induced pluripotent stem cell (hiPSC) lines and two human neural stem cell (hNSC) lines were exposed to a step increase in extracellular glutamine concentration.” The details on cell lines are provided on lines 423-440 in “Materials and Methods”. The authors have also replaced the term “technical replicates” in line 119 for “technical sampling replicates”. The authors have also added to line 445 “(4 bioreactors in total)” to make it clear that two bioreactors of difference cell lines were performed for each cell type.

5 – My interpretation of the response to point 28 is that where data were lost for a given timepoint this was excluded, but the other time points for the same sample were not. This is not appropriate, particularly given the conclusions around dynamicism, as each timepoint should be represented by all samples. If not, then the changes over time will not necessarily reflect consistent change (especially when the n number is unclear).

No time-point was ever discarded in this study, we have used dynamic profiles for all metabolites. But for each time-point (of each cell line) three sampling replicates were collected. The variability between these three sampling replicates reflect technical variations during sample collection/preparation/analysis, not the variability in the metabolic dynamics as all three samples are collected from the same bulk. Therefore, to exclude the potential confounding effects of these unwanted technical variations, we have inspected the relative standard deviation (standard deviation divided by average; RSD) within the three sampling replicates for each metabolite (and for each time point), which allowed us to exclude sampling replicates that were systematically divergent from the other two replicates.

As an example to better explain the pre-processing of samples (referred also in lines 143-157 in manuscript): in hiPSC 1, time-point 30 minutes after glutamine perturbation, RSDs above 10% for all 201 metabolites are related to the fact that one of the replicates is consistently deviating the average for all metabolites. After identifying the deviating replicate – replicate #1 - , new averages and RSDs are determined with the remaining two replicates (replicate #2 and #3) for all 201 metabolites. If those new RSDs are now below 10% for all metabolites, it confirms that the excluded replicate (replicate #1) had some problem during metabolomics processing and is considered an outlier. So, in the end, in hiPSC 1, time-point 30 minutes, one technical sampling replicate (out of three) was removed, meaning that this time point is now represented by two (not three) sampling replicates for the subsequent data analysis. Also, as this cell line has 24 samples in total (8 time-points multiplied by three replicates per time-point), there are still 23 samples used for the study in that cell line and no time-point was discarded.

In the complete dataset, we discarded one of the three sampling replicates in two time-points for hiPSC 1, two for hiPSC 2, one for hNSC 1 and two for hNSC 2. Meaning that 7 out of 96 samples were discarded from the study, which comprises only 7.3% of the complete dataset. See also lines 152-153 in the manuscript.

6 – I was not questioning the validity of the method in 29, rather that quantitative conclusions were being made from qualitative data. The issue with the visibility of the images for publication remain. I printed the images in colour, but they will not print well for those that only use B&W (nor do they reproduce well on screen for the reader to discern the staining).

The authors acknowledge the reviewer’s suggestion, agreeing that a quantitative method for viability analysis could add for clarity of data in a printed version of the manuscript. However, we reason that the image-based qualitative method applied here is sufficient to point out that cell viability is not an issue in this system, as it is evident by Fig 1A where PI-positive cells are negligible. Therefore one can argue that these should not lead to confounding effects on the metabolomics study.

Regarding the visibility of the images and the possible print in B&W, the authors have used the PACE software suggested by the journal to test images’ quality.

7 – Point 32. By 'specific' dynamics I was referring to metabolites.

“Specific dynamics” is not mentioned in point 32 but the authors reckon the reviewer wanted to refer to point 31.

The authors agree that beyond identifying the metabolites having cell-type specific dynamics, the “reason” or some context for those metabolites having that property needs to be provided. With that in mind, the authors have given some examples of previous studies that have identified some of the same metabolites as important regulators – lines 385-391. Implications of finding the class of amino acids as particularly rich for cell-type specific dynamics were detailed next in lines 391-398.

8 – ROCK inhibitor use has become standard, despite little examination of physiological effects. There are however data showing that ROCKi alters metabolism at 24-72h post passaging. What baseline data have the authors generated to confirm that they do not see this change?

This is indeed a relevant topic for all the community, since as the reviewer mentioned there is evidence on the effect of ROCK inhibitor on cell metabolism. This includes a recent paper by Vernardis et al [1], where the authors observed that continuous exposure to 10 μM of Y-27632 leads to metabolic fluctuations of hPSC, although no differences could be observed in gene expression and protein levels of pluripotency and stemness markers. Establishing an accurate baseline to avoid confounding effects of ROCK inhibitor is not trivial. As the use of this small molecule is absolutely necessary for maintaining cell viability, our approach to minimize its impact was to use a lower concentration (5 μM) that is then gradually diluted by the perfusion system (rate of 1.3 day^-1^ or 0.33 day^-1^, for hiPSC and hNSC, respectively). As the perturbation experiment was only performed 2 or 3 days after bioreactor inoculation, that means an estimated ROCK inhibitor concentration of 0.1-0.4 μM for hiPSC and for hNSC.

References:

1.	Vernardis SI, Terzoudis K, Panoskaltsis N, Mantalaris A. Human embryonic and induced pluripotent stem cells maintain phenotype but alter their metabolism after exposure to ROCK inhibitor. Sci Rep. Nature Publishing Group; 2017;7: 1–11. doi:10.1038/srep42138
